# Effects of Celebrity Characteristics, Perceived Homophily, and Reverence on Consumer-Celebrity Para-Social Interaction and Brand Attitude

**DOI:** 10.3389/fpsyg.2021.711454

**Published:** 2021-09-24

**Authors:** Ke Zhang, Menghan Zhang, Chao Li

**Affiliations:** School of Communication, Soochow University, Suzhou, China

**Keywords:** homophily, brand attitude, source attractiveness, source expertise, reverence, para-social interaction

## Abstract

This research explored how perceived homophily and reverence of consumers bridge the gap between endorser characteristics and consumer-celebrity para-social interaction (PSI). Online surveys were utilized to collect data from consumers. The results based on structural equation modeling showed that the perceived attractiveness and expertise of a celebrity were separately antecedent to the perceived homophily and reverence of consumers for the celebrity. This in turn allowed consumers to build PSI with the celebrity and led to a positive attitude toward the celebrity-endorsed brand. No differences were found between non-fans and fans of the selected celebrity regarding the confirmed path from celebrity characteristics to consumer brand attitude *via* PSI and its influencing factors. This work highlighted the significance of consumer-celebrity relations for endorsement effectiveness through proving consumer-celebrity PSI and its drivers as indispensable steps in the endorsement process.

## Introduction

Brand value lies in the emotional connections between consumers and the brand (Zhang, 2010). A common way of creating such brand value is celebrity endorsement. Emphasizing the consumer–celebrity relationship (e.g., para-social interaction, PSI) in the endorsement process essentially refers to attaching importance to the psychological and emotional pleasure and satisfaction that consumers can obtain by consuming a brand endorsed by the celebrity (e.g., Hung et al., 2011; Chung and Cho, 2017).

Para-social interaction refers to the imaginary interaction of audiences with media figures (Horton and Wohl, 1956). PSI manifests the emotional intimacy of consumers with a celebrity endorser, and scholars (e.g., Bao and Dong, 2010; Sha and Zhou, 2013; Centeno, 2016; Escalas and Bettman, 2017) have paid attention to the effects of PSI on the effectiveness of persuasion. Most previous studies have supported that source attractiveness and source expertise are two critical factors affecting the effectiveness of endorsement as well as the strength of consumer-celebrity PSI in the endorsement process (e.g., Conway and Rubin, 1991; Baliantine and Martin, 2005; Schiappa et al., 2007; Hartmann and Goldhoorn, 2011). However, whether celebrity characteristics, including attractiveness and expertise, suffice to enhance the attitude of consumers toward the brand endorsed by the celebrity is not clear and an important question. Are the attractive or professional symbols in the image of celebrities directly stimulating consumers to have a favorable impression on the brands endorsed by celebrities, or are other intermediate factors necessary in linking the image of celebrities with brand attitudes of consumers? The desire of consumers for cognitive and emotional interactions with endorsers, which is closely related to the source characteristics of the celebrity endorser, are factors that researchers and marketers should consider.

The two factors of homophily and reverences, reflecting the cognitive and emotional connection of consumers to the celebrity, are most likely predictors of consumer–celebrity PSI (e.g., Moyer-Gusé, 2008; Brown, 2015). Attitudinal homophily refers to perceived similarities in personality, thinking, values, ways of treating other people, and behavior styles between audiences and celebrities (Tellis, 2004; Bao et al., 2011). In this paper, we argue that celebrity attractiveness affects attitudinal homophily. Reverence refers to the feeling of respect and admiration of audiences for a celebrity (Hoffner, 1996; Belch and Belch, 1999). In this paper, we argue that celebrity expertise affects reverence. We also argue that celebrity attractiveness and expertise have no effect on PSI/brand attitude unless they also lead to homophily and expertise. In other words, celebrity characteristics do not affect the main outcomes (consumers' brand attitudes) unless they also affect the intermediate variables (homophily and reverence).

However, there are a limited number of studies (e.g., Djafarova and Rushworth, 2017; Sokolova and Kefi, 2019) examining homophily, especially in the context of celebrity endorsement. Also, few studies utilize reverence to predict PSI because thinking set in awe of someone is unlikely to promote intimacy in a relationship. Homophily and reverence are two variables closely related to source attractiveness and source expertise, and source attractiveness and source expertise are two significantly predictive factors in celebrity endorsement. With these in mind, this work explored the specific roles of homophily and reverence (both related to source characteristics and consumer–celebrity relations) in the endorsement process. Under what circumstances do source attractiveness and expertise enhance brand attitude in celebrity endorsement? More specifically, what are the roles of homophily, reverence, and PSI? These knowledge gaps need to be clarified.

## Theoretical Framework And Hypotheses

### Celebrity Characteristics and Consumer–Celebrity PSI in Celebrity Endorsement

Brand attitude refers to the overall evaluation of the consumers about the brand (Faircloth et al., 2001; Park et al., 2010; Pina et al., 2019). Sengupta and Fitzsimons (2000) defined brand attitude as psychological evaluation of consumers about brand features and attributes based on previous experience and information, as well as behaviors of consumers influenced by personal cognitive judgment and emotional attribution. Brand attitudes of consumers are closely related to cognitive and emotional satisfaction of consumers (Ha and Perks, 2005; White and Yu, 2005; Oliver, 2014).

The significant influence of source attractiveness and source expertise on brand attitudes of consumers can be found in many advertising studies (e.g., Till and Busler, 2000; Biswas et al., 2006; Amos et al., 2008). Source attractiveness contains three dimensions of physical, social, and task attractiveness in interpersonal communication (McCroskey and McCain, 1974). As perceived intimacy of consumers with a celebrity is an imaginary relation, the physical attractiveness based on the external image of the celebrity, in comparison with the social and task attractiveness based on the celebrity's personality, is easier to gauge by consumers and is the most widely used factor in predicting the effectiveness of endorsement (Ohanian, 1990). Advertisers use physically attractive celebrity endorsers to give consumers sensory stimulation, creating an atmosphere based on the assumption that consumers believe that if they purchase products endorsed or used by these celebrities, they would acquire similar prized attributes (Till et al., 2008).

Using a professional person as an endorser can ensure source credibility, as consumers seem to rely on expert opinions. The results show that an endorser with high professional knowledge in a certain field is more likely to be recognized by consumers than an endorser whose expertise in his or her field is not so convincing (Buhr et al., 1987).

In addition to the power of celebrity characteristics in predicting endorsement effectiveness, the perceived relationship of consumers with the celebrity has been regarded as an increasingly important factor in affecting the attitude of the consumer toward the celebrity-endorsed brand (e.g., Chan and Prendergast, 2008; Hung, 2014; Chung and Cho, 2017). PSI focuses on the inner needs of audiences to achieve psychological and emotional satisfaction by constantly exposing them to celebrities who can provide them with a sense of intimacy and friendship (e.g., Sood and Rogers, 2000; Giles, 2002; Hartmann and Goldhoorn, 2011). Many studies (e.g., Sha and Zhou, 2013; Centeno, 2016; Escalas and Bettman, 2017; Jia et al., 2017) suggest that high degrees of consumer–celebrity PSI intensify the persuasiveness of celebrities in endorsements.

Para-social interaction is a general experience that every individual may participate in and has the function of interpersonal communication (Rubin et al., 1985). PSI is a goal-oriented, positive behavioral trait of audience members that may lead to dysfunction only in extreme cases of excessive obsession (Grant et al., 1991; Schramm and Hartmann, 2008).

Perceived attraction has been supported to be a powerful predictor of PSI (e.g., Conway and Rubin, 1991; Schiappa et al., 2007; Hartmann and Goldhoorn, 2011; Brown, 2015). Although there are few studies directly testing the effect of expertise on consumer–celebrity PSI, some references (e.g., Duran and Kelly, 1988; Wheeless and Reichel, 1990; Turner, 1993; Belch and Belch, 1999; Baliantine and Martin, 2005) have explored the impact of professional skills or knowledge on perception of PSI with media personalities. Generally, source attractiveness and expertise are drivers for perceived PSI of audiences with media figures and predictors of brand attitudes of consumers toward the brand endorsed by the celebrity. Hence, we propose hypothesis 1:

H1: Consumers' perceived PSI with a celebrity plays a mediation role in the relationship between celebrity characteristics, namely, attractiveness (H1a), expertise (H1b), and consumer's brand attitude.

### Homophily and Consumer–Celebrity PSI

Homophily is a perceived similarity rather than an objective similarity between an audience and a celebrity. It emphasizes the similarity of two parameters, namely, attitude and background, between the receiver and source (Gudykunst, 1985; Allen and Post, 2004; Wright, 2004). Background homophily refers to similarities in social class, economic situation, geographic region, and childhood experiences between two individuals. Attitudinal homophily refers to similarities in personality, thinking, values, ways of treating other people, and behaviors between two individuals. The more the audiences recognize that a celebrity is similar to them, the more they attempt to know more about the celebrity (Tellis, 2004; Igartua and Barrios, 2012). Studies (e.g., Cohen, 2004; Cheshire, 2012; Himelboim et al., 2013) have shown that people tend to associate with “like-minded” people. The group identity of these people contributes to willingness of an individual to communicate with other group members.

Previous studies view the two predictors of PSI—homophily and attractiveness—as unrelated to each other. For example, Garrison et al. (1981) found that source attraction and homophily are both powerful predictors for intimating interpersonal relations among friends, acquaintances, and family relatives. Bao et al. (2011) pointed out that the homogeneity between audience and media personae, primarily the similar attitudes, appearance, and background, positively affects the power of PSI. Sokolova and Kefi (2019) confirmed that PSI between influencers and their followers is positively related to the physical attractiveness of influencers and the perceived attitudinal homophily of followers.

However, it may make sense to view homophily and attractiveness as interactional. Celebrities are often regarded as reference groups (Escalas and Bettman, 2003). Consumers prefer the ad with the celebrity whose image is closer to their ideal self-image (Choi and Rifon, 2012). The process of endorsement helps to convey symbolic meaning created by features of celebrity to consumers so that consumers can reconstruct themselves (Escalas and Bettman, 2017). On the one hand, people tend to ascribe other positive attributes, such as good moral values and intelligence, to beautiful people. People with a positive self-image likely see themselves as celebrities—the more they feel like celebrities, the more attractive celebrities are to them. On the other hand, people think that a celebrity looks good, indicating that they are affirmative of the aesthetic level of the celebrity, which not only reflects the aesthetic level of celebrity (the external image of the celebrity is deliberately presented to the public through packaging), but also reflects the aesthetic standards of the audiences. People sharing the same aesthetic standards are more likely to feel that they are the same kind of people (at least in aesthetic attitudes). In short, the more beautiful the endorser, the more similar to them the consumer would feel because a beautiful endorser corresponds more with the ideal self-image of consumers.

Both attitudinal and background homophily have a predictive effect on interpersonal interaction (Moyer-Gusé, 2008; Schmid and Klimmt, 2011; Brown, 2015). There is a low likelihood that ordinary people will have similar backgrounds to celebrities in terms of economic status and social class since celebrities tend to have much higher incomes and reputations; therefore, attitudinal homophily between audiences and celebrities may be more likely to exist than background homophily. For example, Prisbell and Andersen (1980) claimed that attitudinal homophily could reduce uncertainties and improve feelings of safety in a relationship. Gudykunst (1985) suggested that attitudinal similarity leads people to interact and self-disclose to reduce uncertainties between each other. A study by Turner (1993) supported that attitudinal homophily has strong predictive power for PSI between audiences and television performers. Prisbell (1999) confirmed that people in a close relationship such as friendship tend to perceive greater homophily and fewer uncertainties than those in distant relationships.

Generally, the perceived homophily of a consumer with a celebrity seems to be closely related to the attractiveness of the celebrity and might be a driver for consumer–celebrity PSI. Hence, we propose hypotheses 2 and 3.

H2: The attractiveness of the celebrity enhances the consumer's perceived homophily with the celebrity.
H3: Consumer's perceived homophily with celebrity enhances the consumer–celebrity PSI.

### Reverence and Consumer–Celebrity PSI

As audiences are satisfied by the entertainment function of exposure to media figures (Valkenburg and Van der Voort, 1994) and by the inspiration from celebrities, especially those who display laudable characteristics and/or outstanding performance in specific fields (Vorderer et al., 2004; Hung, 2014), the professional capability or perfect personality presented onscreen by a celebrity motivates audiences to follow the behaviors of that celebrity. An individual's desire and need to be a distinguished person can be conveyed by images such as heroes in films and television, athletes who win honor for their country, and talented people from all walks of life (Hung, 2014). This is a process of establishing an ideal image for the individual (Escalas and Bettman, 2003; Choi and Rifon, 2012).

Celebrities are not only objects of entertainment, adoration, and admiration for audiences. These feelings of fondness and admiration may help audiences build a sense of reverence and respect for celebrities. Audiences who regard celebrities as idols and who try to emulate those celebrities (McCracken, 1989; McCutcheon et al., 2002) may continuously engage and maintain relationships with celebrities and also invest energy and money in celebrities (Holt and Thompson, 2004). Advertisers like to use professional athletes to endorse sports products because consumers' sense of respect for these sportsmen will lead to their trust in the quality of the products approved by the sportsmen (Crisci and Kassinove, 1973). Hoffner (1996) supported that the wisdom and power of media figures can bring a strong PSI perception to audiences, especially male audiences, and thus explain the incentive effect of elite images on audiences. Belch and Belch (1999) found that some consumers regard sports celebrities as heroes and psychologically associate the symbol of sports heroes with the brand image that the hero represents.

Meanwhile, celebrities are often regarded as reference groups (Escalas and Bettman, 2003) with significant functions: standard and comparison. The former is to establish a certain measure of behaviors and make the audience comply with it (Kamins, 1990). Celebrities play a normative role for audiences. Audiences consciously follow or internalize the beliefs and values of reference groups of celebrities to keep consistent with them in behaviors. The latter refers to the standard of comparison and starting point for audiences to evaluate themselves or others (Muniz and O'guinn, 2001). For many people, celebrities represent an idealized lifestyle, and when audiences express themselves, they may take the attitude and behaviors of a celebrity as the reference and imitation (Escalas, 2004). Taken together, the perceived reverence of a consumer for a celebrity is closely related to the expertise of the celebrity and may be a driver for consumer–celebrity PSI. Hence, the following hypotheses are proposed.

H4: The expertise of a celebrity enhances the consumer's perceived reverence for the celebrity.
H5: Consumer's perceived reverence for a celebrity enhances consumer–celebrity PSI.

It has been confirmed that the attractiveness and expertise of the source can separately arouse audiences' sense of homophily or reverence for a celebrity (e.g., McCutcheon et al., 2002; Tellis, 2004; Igartua and Barrios, 2012). Meanwhile, homophily and reverence might be predictors of audience–celebrity PSI (e.g., Holt and Thompson, 2004; Vorderer et al., 2004; Moyer-Gusé, 2008; Schmid and Klimmt, 2011). Even the mediating role of homophily and reverence between source attractiveness/expertise and PSI has not been tested. We would like to know what specific role they play when the three factors of source characteristics, homophily/reverence, PSI are put together in a complete endorsement process. Do celebrity characteristics directly affect PSI or must they be mediated by homophily/reverence? With this question in mind, we propose the hypothesis:

H6: Consumer's perceived homophily with (H6a)/reverence for (H6b) a celebrity separately plays a mediation role in the relationship between attractiveness/expertise of celebrity and consumer–celebrity PSI.

Para-social interaction is considered to be a universal emotional connection process between audiences and media figures (Rubin et al., 1985), and audiences' emotional attachment to celebrities is a normal phenomenon reflecting individual identity (Stever, 2011). We suggest that the endorsement process from source characteristics toward brand attitude of consumers *via* PSI might be applied to all consumers, not just for celebrity fans. Therefore, we propose the last hypothesis:

H7: The suggested path from source characteristics (namely, attractiveness and expertise), to PSI and its drivers (namely, homophily and reverence), and then to brand attitude applies to both celebrity fans and non-fans.

**Figure 1:**
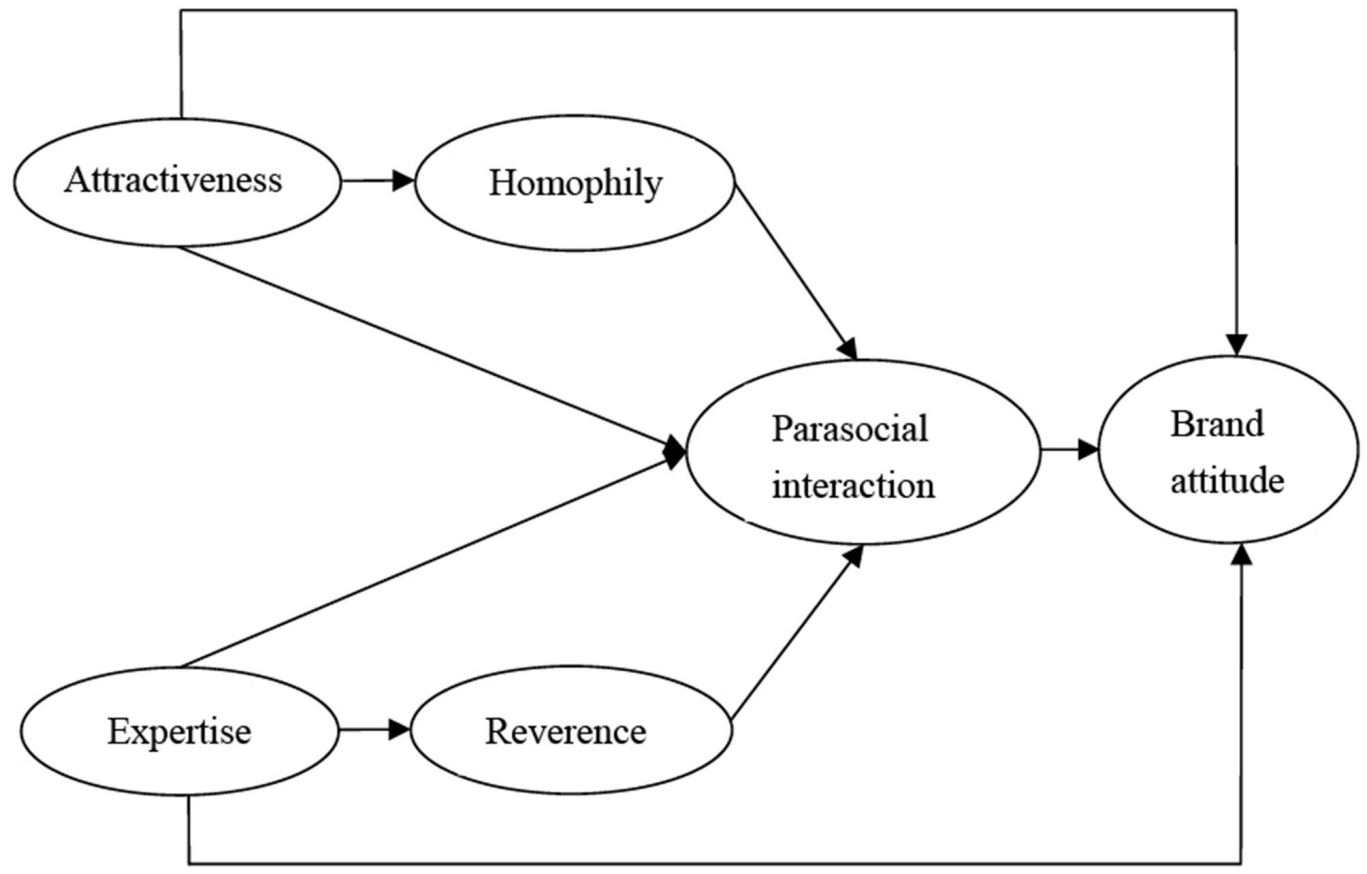
Conceptual model of the endorsement path.

According to the hypotheses, the conceptual framework of the work is portrayed in Figure 1.

## Research Design

The study was conducted in the Chinese market. The Asia-Pacific region remains the world's second-largest advertising market (after North America) and well ahead of Europe, the Middle East, and Africa. China's national advertising market reached $84 billion in 2020, making it the world's second-largest national advertising market (Magna Global, 2020). The vast potential of China's advertising economy makes Chinese consumers a group that cannot be ignored in celebrity endorsement research, and the considerable proportion of China's national advertising market in the world's advertising economy makes the study of China's endorsement phenomenon representative.

### Measures

The measurement instruments used in this study were adapted from the established scale. Ohanian's (1990) measures were used to assess Celebrity Attractiveness and Celebrity Expertise. Attitudinal Homophily was adapted from McCroskey et al. (2006). Reverence was adapted from Mealy et al. (2006). PSI was adapted from Rubin et al. (1985). The brand attitude was adapted from Torres et al. (2007). Table 1 shows the specific measurement items, reliability, and validity.

**Table 1:** Measurement items and reliability and validity assessment.

	**SFL**
*Attractiveness:* Cronbach's α = 0.780; CR = 0.779; AVE = 0.468	
1. I think LT is Attractive	0.704
2. I think LT is Good looking	0.652
3. I think LT is Classy	0.680
4. I think LT is Elegant	0.699
*Expertise:* Cronbach's α = 0.726; CR = 0.725; AVE = 0.398	
5. I think LT is an Expert	0.624
6. I think LT is Experienced	0.595
7. I think LT is Qualified	0.637
8. I think LT is Skilled	0.664
*Para-social Interaction: *Cronbach's α = 0.888; CR = 0.889; AVE = 0.406	
9. I like to watch LT on media.	0.592
10. If LT appeared on a (different) television program, I would watch it.	0.582
11. When LT joins an interview, she seems to understand what I want to know.	0.650
12. When LT shows me how she feels about something, it helps me make up my mind about the issue.	0.699
13. I sometimes compare what LT has said on media with my ideas.	0.633
14. LT makes me feel comfortable as if I am with friends.	0.615
15. If there is no news about LT on the media for a while, I will miss her.	0.605
16. If there are information, comments and story about LT on media, I will read it.	0.700
17. I sometimes make comments on programs that LT participates in.	0.681
18. If I hear words of other athletes different from LT, I will be not satisfied.	0.600
19. I feel sorry for LT when she makes a mistake.	0.644
20. I would like to meet LT in person.	0.589
*Homophily:* Cronbach's α = 0.805; CR = 0.806; AVE = 0.455	
21. I think LT is similar to me in ideas and concepts.	0.620
22. I think I have something in common with LT.	0.678
23. I think LT is similar to me in personality.	0.694
24. I think LT is similar to me in way of speaking.	0.684
25. I think LT is similar to me in treating others.	0.693
*Reverence:* Cronbach's α = 0.806; CR = 0.807; AVE = 0.459	
26. I respect LT.	0.703
27. I think highly of LT.	0.751
28. I dream of becoming someone like LT.	0.579
29. LT is my role model in my career and life.	0.753
30. In my eyes, LT doesn't make mistakes easily.	0.579
*Brand attitude:* Cronbach's α = 0.830; CR = 0.831; AVE = 0.496	
31. I think the brand endorsed by LT is good.	0.640
32. I like the brand endorsed by LT.	0.670
33. I would like to try the brand endorsed by LT.	0.735
34. I will give the brand endorsed by LT priority when consuming.	0.727
35. I would like to recommend others to try the brand endorsed by LT.	0.744

*Alpha refers to Cronbach's alpha reliability estimate; CR refers to composite reliability estimate; AVE refers to average variance extracted; SFL refers to standardized factor loading*.

The demographic information included age, gender, and educational level. Moreover, the questionnaire included a statement to determine whether the respondent was a fan of the chosen celebrity or not. The statement was “I think I am a fan of the celebrity (Liu Tao).”

### Selection of Celebrities

A pretest was made to select a celebrity who meets the source credibility in terms of both attractiveness and expertise. We started with the top 20 highest-paid celebrities in China in 2017 posted by Forbes (2017). Earnings rankings allowed us to include celebrities who were highly involved in endorsements, as endorsements are often a significant part of a celebrity's earnings.

We sent the list to 40 respondents (20 female and 20 male, aged 22–51). They were asked to choose from the list of 20 celebrities who were considered both attractive and expert. The respondents worked independently. According to the results, Liu Tao (ranked 6th on the 2017 Forbeschina list) received the highest number of votes (11 votes), followed by Jacky Cheung (9 votes). Other polls were very diverse. Thus, Liu Tao (LT), an actress who served as an endorser in many advertisements (MRCJCN, 2016), was selected. She had the sixth-highest income according to the list.

### Data Collection

The online questionnaire was placed on the WJX, the largest non-proprietary Chinese online survey platform, with 123 million users collecting 9.792 billion questionnaires by the end of June in 2021 (Official site of WJX, retrieved from https://www.wjx.cn/), during 3 weeks in December 2020. The link to the questionnaire was available to all the sample resources on WJX through both computers and smartphones. Employing convenience sampling, the researchers published the questionnaire link on their WeChat Moments, allowing the questionnaire to be filled in and forwarded. Users with the same IP address could access the questionnaire one time only to avoid repeated responses. The researchers conducted a pretest of the questionnaire using the first 50 responses and fine-tuned the wordings.

A total of 549 respondents joined the survey. At the beginning of the questionnaire, the respondents were provided with the instructions that this survey aimed to have a knowledge on respondents' opinions on a famous Chinese actress Liu Tao and her related things. During the data-cleansing process, respondents who answered the same throughout the questionnaire (e.g., all “3s”) were deleted, as suggested by Meade and Craig (2012). A final sample of 510 respondents was obtained. The effective rate of the questionnaire was 92.9%. The respondents had a balanced gender ratio with 52.2% female (*n* = 266) and 47.8% male (*n* = 244). Those aged 18–30 occupied about two-thirds of the sample population. Nearly 80% of respondents had a University degree or above. About half (51.4%, *n* = 262) of the sample were self-proclaimed fans of LT. The sample demographics are shown in Table 2.

**Table 2:** Demographic characteristics of sample (*N* = 510).

**Variable**	**Item**	**Frequency**	**Ratio (%)**
Gender	Female	266	52.2
	Male	244	47.8
Age	Under 18	23	4.5
	18–30	345	67.6
	31–43	94	18.4
	44–56	45	8.8
	Above 56	3	0.6
Education	Below Bachelor	103	20.2
	Bachelor	340	66.7
	Above bachelor	67	13.1
Fandom	Fans of LT	262	51.4
	Non-fans of LT	248	48.6

## Results

Structural equation modeling (SEM) was used to test the hypotheses. SEM is one of the most commonly used modeling statistics in social science research (Singh, 2009) because it can test a series of correlations simultaneously. Mplus (version 7) was used to conduct a two-step SEM (Kline, 1998) testing the overall path from celebrity characteristics to brand attitudes *via* homophily and reverence and PSI. In the first step of the measurement phase, the work analyzed 35 measurement items and examined the correlated residuals and cross-loadings for each item in order to confirm that they could be combined into indices (the model variables) in accordance with the original measurement scales.

In the second step, confirmatory structural equation model was used to test the relationship between variables. The resulting model had good fit: chi-square value X^2^ (550) = 1252.503; root mean square error of approximation (RMSEA) = 0.050; comparative fit index (CFI) = 0.909; non-normed fit index (NNFI/TLI) = 0.902; root mean square residual (SRMR) = 0.054, in accordance with Bagozzi and Yi (2012). Table 3 shows the correlations between the constructs.

**Table 3:** Variable correlations.

**Variable**	***M (SD)***	***AT***	***EX***	***HO***	***RE***	***PSI***
Attractiveness (AT)	4.00 (0.837)					
Expertise (EX)	3.78 (0.844)	0.228^*^				
Homophily (HO)	4.03 (0.568)	0.485^**^	0.301^*^			
Reverence (RE)	3.93 (0.631)	0.328^*^	0.562^**^	0.390^*^		
Para-social interaction (PSI)	3.51 (0.880)	0.593^**^	0.564^**^	0.643^**^	0.425^**^	
Brand attitude (BA)	3.63 (0.931)	0.511^**^	0.322^*^	0.564^**^	0.370^**^	0.636^**^

*N = 510; the variable correlations are shown in Pearson's r values*.

* *means the correlations are significant at p, 0.5;*

** *means the correlations are significant at p, 0.01*.

### Hypothesized-Path Model Test

We first tested the correlations among variables to ensure their divergent validity. Table 3 shows that celebrity physical attractiveness and expertise were fairly independent (*r* = 0.228), as were homophily and reverence (*r* = 0.390). Meanwhile, the correlations between attractiveness–homophily and expertise–reverence are moderate (*r* about 0.500–0.600). These indicate that the two groups of variables (attractiveness and its related homophily vs. expertise and its related expertise) belong to two parallel branches influencing PSI. In addition, both homophily (*r* = 0.643) and reverence (*r* = 0.425) have moderate correlations with PSI, indicating that homophily and reverence may have the potential to be predictors of PSI. The influencing effects were tested below.

**Figure 2:**
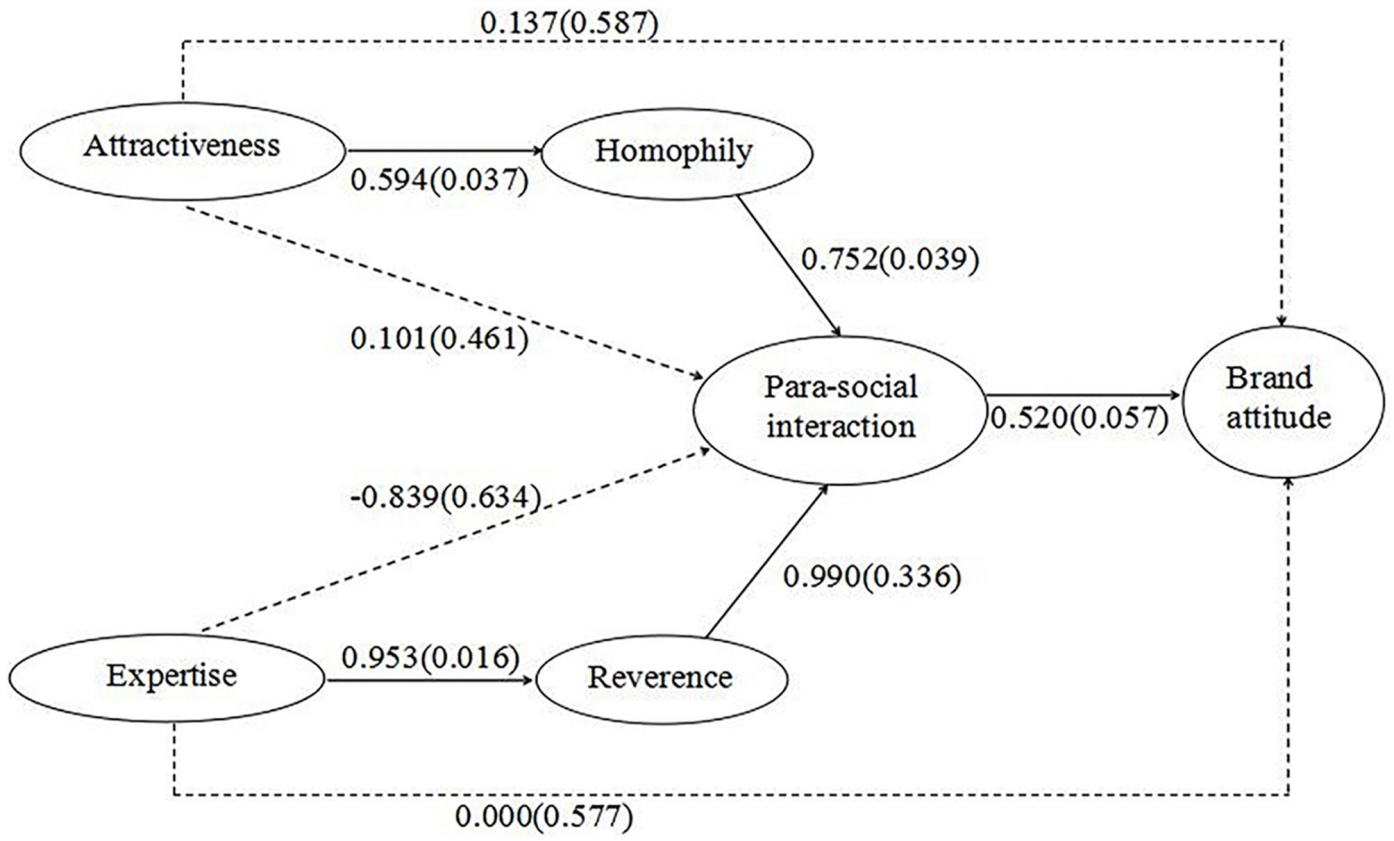
Statistical model of the endorsement path. The standardized coefficients (with stand error in parentheses) of the solid paths are significant at *p*, 0.01; Model fit: Chi-square value X^2^(550), 1252.503, RMSEA, 0.050, CFI, 0.909, TLI, 0.902, SRMR, 0.054.

The SEM simultaneously tested H1–H6, where support for H2–H5 together partly supported H6, taken together forming the basis for testing H1. Figure 2 shows the results of the standardized model.

First, the lack of any direct relation between celebrity attractiveness and brand attitude (β = 0.137, *p* = 0.815), and the lack of any direct relation between celebrity expertise and brand attitude (β = 0.000, *p* = 1.000), together with the support for H2–H6 (see below) supported H1: PSI mediates the relation between source characteristics and brand attitude.

In support of H2, celebrity attractiveness had a significant relation to homophily (β = 0.594, *p* = 0.000), which in turn had a significant relation to PSI (β = 0.752, *p* = 0.000), supporting H3. Similarly, in support of H4, celebrity expertise had a significant relation to reverence (β = 0.953, *p* = 0.000), which in turn had a significant relation to PSI (β = 0.990, *p* = 0.003), supporting H5. Together with the findings that there was no direct relation between celebrity attractiveness and PSI (β = 0.101, *p* = 0.826) or between celebrity expertise and PSI (β = −0.839, *p* = 0.186), this supported H6: Homophily and reverence separately mediate the relation between source attractiveness or expertise and PSI.

Another test to establish mediators is that the confidence interval of summary path effects should not include zero. Using the bootstrap syntax, model indirect, and cinterval directives in Mplus (Muthén and Muthén, 2017), it was found that neither the path from celebrity attractiveness through homophily and PSI to brand attitude (lower 2.5% limit = 0.148, upper 2.5% limit = 0.308), nor the path from celebrity expertise through reverence and PSI to brand attitude (lower 2.5% limit = 0.344, upper 2.5% limit = 0.516) contained zero, lending further support to H1a–b.

Meanwhile, it was found that neither the path from celebrity attractiveness through homophily to PSI (lower 2.5% limit = 0.344; upper 2.5% limit = 0.516), nor the path from celebrity expertise through reverence to PSI (lower 2.5% limit = 0.149; upper 2.5% limit = 0.351) contained zero, lending further support to H6a–b. Table 4 shows the output files containing the estimated mediation effects and details of the BC boost confidence intervals.

**Table 4:** Output file of confidence intervals of standardized mediation effects.

	***L*.5%**	***L* 2.5%**	***L* 5%**	**Estimate**	***U* 5%**	***U* 2.5%**	***U*.5%**
**Confidence intervals of standardized total, total indirect, specific indirect, and direct effects**							
Effects from ATT to PSI							
Sum of indirect	0.316	0.344	0.357	0.430	0.502	0.516	0.543
Specific indirect							
PSI							
HOM							
ATT	0.316	0.344	0.357	0.430	0.502	0.516	0.543
Effects from EXP to PSI							
Sum of indirect	0.117	0.149	0.165	0.250	0335	0.351	0.383
Specific indirect							
PSI							
REV							
EXP	0.117	0.149	0.165	0.250	0335	0.351	0.383
Effects from HOM to BA							
Sum of indirect	0.239	0.275	0.294	0.390	0.487	0.506	0.542
Specific indirect							
BA							
PSI							
HOM	0.239	0.275	0.294	0.390	0.487	0.506	0.542
Effects from REV to BA							
Sum of indirect	0.046	0.069	0.080	0.140	0.200	0.212	0.234
BA							
PSI							
REV	0.046	0.069	0.080	0.140	0.200	0.212	0.234
Effects from ATT to BA							
Sum of indirect	0.123	0.148	0.161	0.228	0.295	0.308	0.333
Specific indirect							
BA							
PSI							
HOM							
ATT	0.123	0.148	0.161	0.228	0.295	0.308	0.333
Effects from EXP to BA							
Sum of indirect	0.045	0.066	0.077	0.133	0.188	0.199	0.220
BA							
PSI							
REV							
EXP	0.045	0.066	0.077	0.133	0.188	0.199	0.220

*ATT, Attractiveness; EXP, Expertise; PSI, Para-social interaction; HOM, Homophily; REV, Reverence; BA, Brand attitude; L, Lower; and U, Upper*.

### Multigroup Analysis

A multigroup analysis was performed using Mplus to verify the difference in the hypothetical path model between fans and non-fans of the selected celebrity. In this analysis, each causal path was constrained equally across the two groups simultaneously, and a model with each constrained path was contrasted against the fully unconstrained model. If the X^2^, AIC, and BIC values of a model with any constrained path became significantly worse than those of the unconstrained model, then the coefficients of the constrained path were identified as significantly different between the two groups (Kline, 1998).

For the information criteria AIC and BIC, lower values indicated an improvement in model fit (Burnham and Anderson, 2004; Vrieze, 2012). The SEM model with constrained regression coefficients was only slightly lower in the model fit information [X^2^ (1167) = 2111.705, AIC = 42181.246, and BIC = 42871.455] than the model with unconstrained regression coefficient [X^2^ (1158) = 2100.568, AIC = 42188.110, and BIC = 42916.428]. The result showed no significant difference between non-fans and fans in generating their attitudes toward the celebrity-endorsed brand followed by the hypothesized-path model. Thus, hypothesis 7 is supported that the endorsement path based on the consumer–celebrity relations could be generalized to both non-fans and fans of the chosen celebrity.

### Summary of Results

The results indicated that source characteristics of a celebrity could affect the brand attitude of consumers only in the case that consumers first established perceived homophily and reverence for the celebrity, successively built PSI with the celebrity, which taken together meant a positive attitude toward the celebrity-endorsed brand. The factors of perceived homophily and reverence of consumers were important variables linking the external merits of endorsers with the internal attachment of consumers.

The confirmed significant effect of perceived homophily toward the brand attitude in this study indicated that the higher the homogeneity between the spokesperson and the consumer, the more interactions, the more effective the endorsement was likely to be. Meanwhile, the supported mediation effect of perceived reverence between celebrity's expertise and consumer–celebrity PSI in this study demonstrated that recognition and respect for an endorser based on professional strength might be one of the motivations to attract consumers to get close to the endorser psychologically.

Furthermore, the proposed path from celebrities' characteristics to consumers' brand attitudes *via* PSI and its drivers was feasible for both fans and non-fans, indicating the path had some generality.

## Discussion

As the consumer–celebrity relationship becomes increasingly crucial to celebrity endorsement (Hung et al., 2011; Chung and Cho, 2017), the emotional needs of consumers have been paid more attention by researchers (Sood and Rogers, 2000; Giles, 2002; Hartmann and Goldhoorn, 2011). The research-supported path placed the relationship of consumers with the endorser at a critical position in the endorsement process, providing more detailed insights for understanding the meaning transferring from the perceived celebrity characteristics to the brand attitude of consumers in the endorsement process.

The perceived attractive appearance or professional ability of a celebrity endorser only changes consumers' brand attitude by consumers' perceived relations with the celebrity (including PSI and homophily/reverence). Celebrity endorsement is not a simple process of consuming the merits or advantages of a celebrity endorser, suggesting that marketers should focus on the capacity of celebrity to arouse emotional attachment of consumers rather than blindly choose a celebrity who is outwardly charming or of high status or prestige.

Although PSI with a celebrity is something that takes place in the imagination of the audiences, it has the power to move the audiences. It is an almost universal feeling to develop a fondness for a celebrity, and this emotional state is very similar to regular interpersonal communications in the real world (Rubin et al., 1985; Grant et al., 1991; Schramm and Hartmann, 2008). Audiences gain psychological and emotional satisfaction by maintaining this imaginary relationship with a celebrity. People's recognition of a celebrity, regardless of being fans or non-fans, improves their appreciation (regarding the celebrity as the same kind of person in some ways) and respect (regarding the celebrity as a role model in life) for the celebrity. Individuals' appreciation and respect are already an inner relationship between individuals and the celebrity, mentioning psychological and emotional identification in connection with the celebrity. It leads consumers to be easily persuaded by the celebrity to accept a specific brand.

The results of using homophily as a predictor of celebrity perception suggest that consumers' sense of similarity to celebrities does close the psychological relationship between consumers and celebrities, which is consistent with the nature of celebrity perception in reducing uncertainty and increasing familiarity (Prisbell and Andersen, 1980; Gudykunst, 1985). People tend to get along with individuals with matching personalities, beliefs, and values (Cohen, 2004). The sense of familiarity, similar to contacting an old acquaintance or friend, makes consumers feel that they are getting along with like-minded people who have a shared identity (Cheshire, 2012; Himelboim et al., 2013). Once this false sense of familiarity has been established, a recommendation from the celebrity as a member of a reference group (Assael, 1984; Kamins, 1990; Escalas, 2004) reduces the consumer's feelings of uncertainty toward the brand. Meanwhile, the complete mediation effect between celebrities' attractiveness and consumer–celebrity PSI suggests that homophily closely related to source attractiveness (Ohanian, 1990; Tellis, 2004) can close the distancing between individuals (Cheshire, 2012; Himelboim et al., 2013) and explain the willingness of a consumer to interact with a celebrity based on the appearance of a celebrity.

Reverence is a driver for PSI, which reveals the basic human behavior of emulating those better than oneself (McCracken, 1989; McCutcheon et al., 2002). As a reference group (Assael, 1984; Escalas, 2004), celebrities usually play an idealized image for ordinary people. On the one hand, audiences may use celebrities to express themselves and improve their self-images. On the other hand, they may be particularly fond of or very loyal to some celebrities and the group and want to maintain a long-term relationship with it, thus regarding the group values as their values.

Consumers tended to show compliance with a celebrity perceived to be highly professional in a particular field. The underlying psychology of reaching the same social or economic status as the celebrity may be the basis for establishing a friendship-like intimacy with the celebrity. It indicates that PSI may not just be a sense of intimacy akin to getting along with a friend, but rather a respect for role models. It enables individuals to project an ideal image in terms of social status, financial ability, and personal taste (Sirgy, 1982; Escalas and Bettman, 2003; Choi and Rifon, 2012). When a person feels that he or she can attain the same level of excellence as that displayed by a reference group, the person is motivated to engage in and maintain a relationship with that reference group (Holt and Thompson, 2004).

## Contributions

The theoretical contribution of the work contains two points. First, the test of PSI and its drivers emphasizes the consumer–celebrity relation. The confirmed path places consumer–celebrity ties at the center of the endorsement process. It is a supplement for the mainstream celebrity-centric perspective in endorsement research. Second, compared with attractiveness and expertise, homophily and reverence are two factors that have not received enough attention in previous studies in celebrity endorsement. Although they are not new variables, few studies utilize the homophily and reverence scales in endorsement. Thus, exploring the effects of perceived homophily and reverence toward brand attitudes can inspire researchers to pay more attention to these variables closely related to consumer–celebrity relations.

For marketers, it is worth considering cultivating a loyal consumer relationship with a brand by utilizing consumers' inner connection with a celebrity based on cognitive and affective communication. The sales of products driven by consumers' consumption behaviors are the ultimate goal of marketers. When consumers establish an emotional connection with a celebrity endorser, they tend to feel favorable toward the brand that the celebrity uses or recommends and are more inclined to believe in the quality of the brands the celebrity endorses. The PSI of consumers and celebrities can be fully expanded through multimedia platforms, including mass media and social media. Especially with the rise and flourishing of social media, there are many ways for consumers to participate in promoting brands and products. Given sufficient celebrity awareness, celebrity endorsement hence seems to be effective also for non-fans, which would be important knowledge for advertisers.

## Limitations And Future Research

Several limitations exist in this study. First, self-reported data collected through surveys cannot account for causality between variables in the model. Experimental tests can be added in future studies to support the results obtained in this study. Second, the brands tested in this study were not specified, which may confuse respondents in reporting their attitudes toward the general brands endorsed by the chosen celebrity or inflate the effect of PSI on brand attitude. Future research should introduce a pretest to select a specific brand with high/low congruence with the celebrity to better assess the magnitude of the effect of PSI on brand attitudes. Besides, only 40 people took part in the pretest, which can affect the representation of the chosen celebrity. More celebrities could be utilized with a stricter selection procedure in such studies. Third, more attention should be paid to demographic factors, thus exploring the connection between consumers and celebrity endorsers. Consumers of different ages may have different motivations and needs when it comes to connecting with celebrities. In this study, young people account for the majority. Further research may look at replicating these tests with a broader range of consumers, celebrities, and specified brands, to test the feasibility of the established model.

## Data Availability Statement

The raw data supporting the conclusions of this article will be made available by the authors, without undue reservation.

## Ethics Statement

The studies involving human participants were reviewed and approved by Committee on the Use of Human & Animal Subjects in Teaching and Research (HASC) of Hong Kong Baptist University. Written informed consent for participation was not required for this study in accordance with the national legislation and the institutional requirements.

## Author Contributions

KZ and MZ contributed to the hypotheses initiation, research design, and manuscript writing. CL contributed to the data collection and analysis. All authors contributed to the article and approved the submitted version.

## Funding

This research was funded by the Social Science Fund of Jiangsu Province Innovation of Video Communication Technology and Integration of Mainstream Media under 5G Conditions (Grant No. 20XWC002) and the 2021 Project of Philosophy and Social Science of Jiangsu Universities Research on the Influence and Regulation of Social Robots on the Climate of False Opinions in International Communication (Grant No. 2021SJZDA151).

## Conflict of Interest

The authors declare that the research was conducted in the absence of any commercial or financial relationships that could be construed as a potential conflict of interest.

## Publisher's Note

All claims expressed in this article are solely those of the authors and do not necessarily represent those of their affiliated organizations, or those of the publisher, the editors and the reviewers. Any product that may be evaluated in this article, or claim that may be made by its manufacturer, is not guaranteed or endorsed by the publisher.
